# From Trauma to Suicidal Ideation in a Spanish Chronic Pain Population: Cognitive Mediation in the Genesis of Psychological Suffering

**DOI:** 10.3390/jcm15020715

**Published:** 2026-01-15

**Authors:** Juan José Mora-Ascó, Carmen Moret-Tatay, María José Beneyto-Arrojo, Miguel Pedro León-Padilla

**Affiliations:** 1 Doctoral School, Catholic University of Valencia San Vicente Mártir, 46002 Valencia, Spain; 2 Department of Personality, Treatments and Methodology, Faculty of Psychology, Catholic University of Valencia San Vicente Mártir, 46001 Valencia, Spain; mariacarmen.moret@ucv.es (C.M.-T.); mariajose.beneyto@ucv.es (M.J.B.-A.); 3 Veritatis Gaudium Institute, Catholic University of Valencia San Vicente Mártir, 46001 Valencia, Spain; miguel.leon@ucv.es

**Keywords:** childhood trauma, chronic pain, suicidal ideation, perceived burdensomeness, hopelessness, pain catastrophizing

## Abstract

**Background:** Chronic pain is consistently associated with increased vulnerability to suicidal ideation, particularly among individuals with a history of early adverse experiences. However, the cognitive mechanisms linking childhood trauma to suicidal thoughts in this population remain insufficiently understood. **Methods:** A cross-sectional study was conducted with 251 adults living with chronic pain. Participants completed validated measures assessing childhood trauma, perceived burdensomeness, hopelessness, pain catastrophizing, and suicidal ideation. Correlational analyses were conducted to examine associations among variables, followed by a multiple mediation model to test the mediating role of cognitive processes. Data were analyzed using Pearson correlations and robust-estimation mediation procedures implemented in JASP. **Results:** Childhood trauma showed positive and significant associations with perceived burdensomeness, hopelessness, pain catastrophizing, and suicidal ideation. Mediation analyses showed that perceived burdensomeness, hopelessness, and pain catastrophizing significantly mediated the relationship between childhood trauma and suicidal ideation, with small-to-moderate indirect effects. These findings suggest that maladaptive cognitive patterns may partially explain how early adverse experiences are associated with suicidal thoughts in individuals with chronic pain. **Conclusions:** The results highlight the relevance of considering early adverse experiences and pain-related cognitive processes in the clinical assessment of suicidal ideation among individuals with chronic pain. Further research using longitudinal and multimethod designs is needed to refine explanatory models and guide psychological interventions aimed at reducing vulnerability to suicidal ideation in this population. This study expands existing knowledge by simultaneously examining perceived burdensomeness, hopelessness, and pain catastrophizing as mediators between childhood trauma and suicidal ideation in individuals with chronic pain. These findings contribute to refining trauma-informed clinical approaches and identifying specific intervention targets.

## 1. Introduction

Suicide constitutes a major global public health concern. According to the World Health Organization [1], approximately 727,000 suicide deaths were recorded in 2021, placing this phenomenon among the leading causes of premature mortality and showing particularly high prevalence among individuals aged 15 to 29 years [2]. Its significance underscores the need to deepen our understanding of the personal factors and psychosocial mechanisms that may precede the onset of suicidal ideation, one of the strongest predictors of suicide mortality [3,4].

Within this context, chronic pain emerges as a clinical condition associated with a markedly increased risk of developing thoughts and behaviors related to one’s own death. Supporting this assertion, a meta-analysis including more than 3 million participants found a pooled prevalence of suicidal ideation of 28.9% among individuals with chronic pain, and a prevalence of suicide attempts of 10.8%. Moreover, recent suicidal ideation (within the past two weeks) reached 25.9% in the analyzed sample. These findings indicate that approximately one in four individuals living with persistent pain may be experiencing thoughts related to the end of life [5].

Similarly, other studies have shown that the detection of suicidal ideation following screening is higher in patients than in the general population, even after controlling for other psychiatric comorbidities. This suggests that pain operates as an independent factor increasing suicide risk [6].

Despite this connection, the direct link between chronic pain and suicidal ideation may be better understood by considering other influential variables, including adverse childhood experiences. Recent literature has shown that early-life difficulties, such as traumatic events involving abuse or neglect, heighten the likelihood of developing primary chronic pain in adulthood [7]. These experiences have also been associated with higher rates of suicidal ideation and suicidal behavior [8]. Supporting this perspective, Bahk et al. [9], in their study with adults, found that childhood trauma directly predicted suicidal ideation. Thus, the interaction between chronic pain and adverse childhood experiences may create a cumulative burden that substantially elevates suicide risk [10].

Given these circumstances, a central question arises in understanding how the psychological interaction between early negative experiences and suicidal ideation unfolds within the context of chronic illness and persistent pain. In this regard, and in line with contemporary models explaining the development of suicide risk [11,12], maladaptive cognitive processes may operate as mediating mechanisms by significantly shaping the way patients interpret and cope with the progressive changes associated with illness and pain [13,14]. Within this cognitive domain, key variables include perceived burdensomeness, hopelessness, and pain catastrophizing, all of which have shown consistent associations with psychological distress and suicidal ideation across various medical conditions [15,16,17].

However, despite increasing evidence supporting the relevance of these cognitive mechanisms, the current literature has not yet examined them jointly as simultaneous mediators between childhood trauma and suicidal ideation in individuals with chronic pain. This absence of integrative models represents a key scientific gap, limiting both theoretical development and the clinical capacity to identify specific cognitive pathways that may inform risk detection and intervention.

Among these processes and considering their particularly relevant role in the subjective experience of persistent pain, it is pertinent to first examine perceived burdensomeness. This construct, formulated within the Interpersonal Theory of Suicide, refers to the belief of being an added responsibility and a hindrance to others, particularly significant individuals who may assume caregiving roles [18]. For this reason, perceived burdensomeness has been identified as a robust predictor of suicidal ideation, as it reinforces the belief that others would be better off if one no longer existed [19]. In the context of chronic pain, such perceptions may be intensified due to functional dependence, reduced productivity, and the need for ongoing support [20].

A second variable of interest is hopelessness, widely characterized in the literature as a cognitive state marked by the perception of a lack of solutions or positive expectations when facing adversity, which leads to feelings of frustration, helplessness, and demotivation [21]. Grounded by these thoughts and emotions, hopelessness emerges as a key risk factor for suicide [22]. Supporting this assertion, Ribeiro et al. [23], in their meta-analysis, concluded that hopelessness doubled the risk of suicide, driven by the loss of future goals and the intensification of suicidal ideation.

Finally, pain catastrophizing is understood, following Petrini and Arendt-Nielsen, as the tendency to magnify the negative implications of pain and to anticipate the worst possible outcomes [24]. In this regard, recent research conceptualizes pain catastrophizing as a fundamental cognitive process in conditions involving persistent pain, linking it to increased psychological distress, poorer functioning, and heightened suicide risk [25]. Sustained negative thoughts about the future fosters the use of maladaptive coping strategies when confronting the challenges posed by illness, thereby increasing pain interference and limiting patients’ functional engagement in daily life [26].

The present study builds on previous evidence highlighting the central role of cognitive processes in the relationship between early adverse experiences and vulnerability to suicidal ideation [27,28]. However, although these studies underscore the relevance of such factors in populations exposed to childhood trauma, none have examined them specifically within the context of chronic pain, pointing to a significant gap in the current literature.

**Figure 1:**
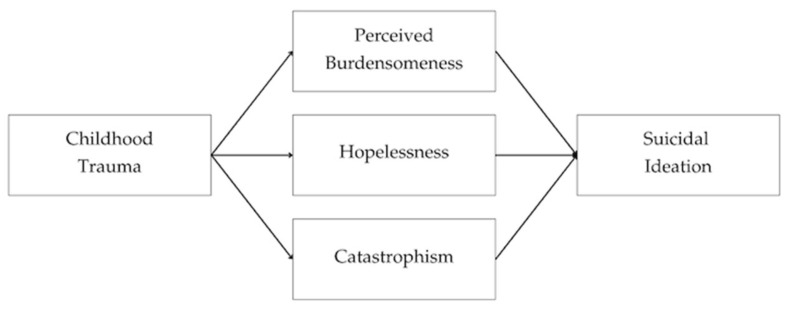
Theoretical mediation model illustrating the role of maladaptive cognitive processes in the relationship between childhood trauma and suicidal ideation.

In this regard, the available research remains limited with respect to the development of models that simultaneously integrate the complexity of the cognitive dimension (including perceived burdensomeness, hopelessness, and pain catastrophizing) as potential mediators between childhood trauma and suicidal ideation in individuals with persistent pain. Examining these mediational pathways jointly allows for a deeper understanding of the etiology of suicidal thoughts in this population, which in turn may serve as a basis for developing more specific strategies for risk detection and early psychological intervention. Along these lines, the present work examines a theoretical model that incorporates the three mediators, thereby synthesizing the hypothesized relationships among childhood trauma, maladaptive cognitive processes, and suicidal ideation (see Figure 1). Given the absence of integrative mediation models in this field, the present study was conceived as exploratory. Therefore, no directional hypotheses were formulated a priori. Instead, the mediational pathways were examined with the aim of informing future theoretical refinement and improving the clinical understanding of suicidal ideation in individuals with chronic pain.

Although the present study focuses on maladaptive cognitive processes, it is acknowledged that adaptive cognitions, such as coping strategies, social support engagement, and self-efficacy, also function as relevant protective mechanisms in chronic pain and suicidality. These constructs are conceptually related to the topic and, although they are not examined empirically in the present model, they represent a complementary dimension that clarifies the specific focus adopted in this study.

Based on this theoretical framework and the needs identified in the existing literature, the present study pursues two primary objectives: (1) to examine the associations among childhood trauma, perceived burdensomeness, hopelessness, pain catastrophizing, and suicidal ideation in individuals with chronic pain, and (2) to analyze the mediating role of these cognitive processes in the relationship between childhood trauma and suicidal ideation through a multiple mediation model.

## 2. Materials and Methods

### 2.1. Participants

The study sample consisted of 251 adults drawn from different autonomous communities across Spain. The largest groups were from Valencia (38.6%) and Madrid (37.5%), followed by smaller proportions from Castilla-La Mancha (6.0%), Murcia (4.8%), Andalucía (4.4%), Catalonia (3.2%), Castilla y León (3.2%), and Galicia (2.0%). Additional participants came from Aragón, Extremadura, the Balearic Islands, Navarre, the Basque Country, and Melilla, each representing less than 1% of the total sample. Participants’ ages ranged from 18 to 84 years (*M* = 50.51, *SD* = 9.93).

In terms of sex distribution, 39 participants (15.5%) were men and 212 (84.5%) were women. Most participants (78.9%) identified as belonging to a middle socioeconomic level, and approximately one third (33.9%) indicated that they were the main financial provider in their household.

Recruitment was carried out through hospital pain units and chronic pain patient associations. Inclusion criteria were: (a) having a medical diagnosis of chronic pain, and (b) being over 18 years of age. Exclusion criteria included severe psychiatric disorders with psychotic features, cognitive impairment that could interfere with the completion of self-report instruments, and active substance dependence. No additional exclusion criteria were applied beyond these thresholds.

Although pain intensity was not used as a formal inclusion criterion, the majority of participants reported high levels of pain (VAS > 7) once recruited, resulting in a sample predominantly composed of individuals experiencing severe pain. This distribution reflects the clinical profile commonly observed in patients diagnosed with chronic pain. The mean pain score for the sample was *M* = 7.95 (*SD* = 1.53), indicating a high level of perceived pain intensity among participants.

### 2.2. Procedures

The study was approved by the University Ethics Committee (Code: UCV/2022-2023/034/v2), and authorization was obtained from the legal representatives of the participating institutions. Five organizations participated in total: three patient associations and two hospital pain units. Recruitment occurred between November 2023 and February 2024. Information about the study was provided to potential participants, and informed consent was obtained prior to inclusion.

Data collection procedures differed according to institutional feasibility. In patient associations, eligible members were contacted via a mass mailing distributed by organizational coordinators and accessed the survey through a secure link. Hospital pain units did not authorize mass electronic distribution, so data collection was conducted in person prior to scheduled clinical appointments. In both contexts, responses were completed individually and autonomously using participants’ own devices (smartphones or personal computers), ensuring comparable survey conditions. All data were collected anonymously, and no identifiable information was recorded at any point.

The questionnaire was administered through Microsoft Forms and included sociodemographic and psychological measures. The final sample consisted of 60 (23.90%) hospital-based respondents and 191 (76.10%) association-based respondents. The same dataset has been used in previous research to examine related psychological and clinical constructs; however, the present study addresses distinct research questions and generates novel findings that had not been analyzed before.

### 2.3. Materials

The selected instruments were prioritized due to their clinical relevance in chronic pain populations, their prior validation in Spanish samples, and their conceptual alignment with the cognitive mechanisms examined in this study.

Individual Models of Relationships Revised (CaMir-R; Balluerca et al. [29]).

The CaMir-R assesses attachment representations and perceptions of family functioning in adolescents and adults. The version used in this study comprises 32 items rated on a five-point Likert scale, organized into seven dimensions: security, family concern, parental interference, self-sufficiency and resentment toward parents, childhood trauma, value of parental authority, and permissiveness. These dimensions enable the analysis of secure, avoidant, preoccupied, and disorganized attachment styles within family relationships.

For the purposes of this study, the childhood trauma dimension was selected due to its theoretical and empirical relevance to suicidal ideation. This subscale captures memories of violence, threat, and parental unavailability, which underlie insecure (anxious and avoidant) and, in some cases, disorganized attachment patterns [29,30]. The Spanish validation [29] reported reliability coefficients ranging from *α* = 0.60 to *α* = 0.85 across subscales. In the present sample, Cronbach’s alpha coefficients exceeded *α* = 0.64, indicating acceptable internal consistency.

The CaMir-R trauma subscale was selected due to its relational and attachment-based orientation, which aligns with the conceptual framework of this study. While classical instruments (e.g., Childhood Trauma Questionnaire) offer a broader taxonomy of adverse experiences, the CaMir-R provides sensitivity to interpersonal dimensions that are theoretically relevant for understanding the cognitive mechanisms examined here. This choice prioritizes conceptual coherence with the study aims, acknowledging that the measure focuses specifically on relational trauma rather than aiming to capture the full spectrum of adverse childhood experiences.

Interpersonal Needs Questionnaire–15 items (INQ-15; Van Orden et al. [31]).

The Interpersonal Needs Questionnaire–15 items evaluate the two core constructs of the Interpersonal Theory of Suicide [32]: perceived burdensomeness and thwarted belongingness, both considered predictors of suicidal ideation and attempts.

The brief version used in this study comprises 15 items rated on a Likert-type scale, distributed into two subscales: perceived burdensomeness (items 1–6) and thwarted belongingness (items 7–15). Psychometric analyses have shown that these constructs are distinct yet reliable factors that can be independently assessed [31,33]. In the present sample, internal consistency was *α* = 0.60 for thwarted belongingness and α = 0.89 for perceived burdensomeness.

Beck Hopelessness Scale (BHS; Beck et al. [34]).

The Beck Hopelessness Scale (BHS) examines the level of hopelessness through negative expectations about the future, perceived personal resources, and the ability to cope with life challenges. It consists of 20 dichotomous items (true/false), grouped into three factors: feelings about the future, loss of motivation, and future expectations. Total scores range from 0 to 20, with four severity levels: minimal (0–3), mild (4–8), moderate (9–14), and severe (15–20).

The Spanish adaptation by Viñas et al. [35] reported an internal consistency of *α* = 0.93, while later studies in Spanish populations with suicide attempts reached *α* = 0.98 [36] In the present sample, reliability for the BHS was *α* = 0.90.

Pain Catastrophizing Scale (PCS; García et al. [37]).

The Pain Catastrophizing Scale is a widely used instrument designed to measure pain coping from a catastrophizing perspective. Its development is grounded in earlier research identifying three central components of the pain catastrophizing response: the tendency to focus on pain-related thoughts, the exaggeration of perceived threat in response to pain stimuli, and the sense of helplessness when facing pain.

The PCS comprises 13 items rated on a 5-point Likert scale ranging from 0 (not at all) to 4 (all the time), distributed across three subscales—Rumination, Magnification, and Helplessness—as well as a total score reflecting the overall level of pain catastrophizing. In the original validation, Sullivan et al. [38] reported internal consistency coefficients of *α* = 0.87 for the total score and *α* = 0.87, *α* = 0.60, and *α* = 0.79 for rumination, magnification, and helplessness, respectively. The Spanish adaptation by García et al. [37] obtained a reliability of *α* = 0.70 for the global score. In the present sample, internal consistency for the total PCS score was *α* = 0.91.

Scale for Suicide Ideation (SSI-HF; Beck et al. [39]; Huamaní & Fuentes [40]).

The Scale for Suicide Ideation (SSI) was developed to evaluate the intensity and characteristics of suicidal thoughts (Beck et al. [39]). The Spanish form adapted by Huamaní and Fuentes [39] includes 16 dichotomous items (yes/no) evaluating key indicators of suicide risk such as desire to die, previous attempts, help seeking, social support, planning, and perceived closeness to death. Affirmative responses are scored as 1 point, except for items 3, 4, 8, and 12, which score 2 points due to their greater clinical relevance. Total scores classify risk as mild (0–5), moderate (6–11), or high (12–20).

Psychometric studies have reported excellent internal consistency (*α* = 0.89; Pulido et al. [41]). In the present sample, the Beck-HF showed a reliability of *α* = 0.84.

### 2.4. Data Analyses

Descriptive analyses were first conducted to characterize the distribution of sociodemographic and clinical variables in the sample. Bivariate associations among the study variables were subsequently examined using Pearson correlation coefficients, a widely accepted procedure for assessing linear relationships between continuous measures.

Robust estimation procedures were applied to address potential deviations from normality, and indirect effects were estimated using bias-corrected 95% bootstrap confidence intervals. A multiple mediation model was used to examine predictive and mediational relationships, ensuring stable parameter estimates and accurate standard errors. No covariates were included in the statistical model; however, the unequal sex distribution of the sample was considered conceptually during analysis to contextualize interpretation without altering the original mediation structure.

Although hospital-based data were collected in person and association-based data online, no substantial differences were observed in descriptive indicators between recruitment contexts. Formal equivalence testing was not conducted, but demographic and clinical patterns were comparable, supporting interpretive consistency across data collection settings.

All statistical analyses were performed using JASP (version 0.19).

## 3. Results

Firstly, preliminary analyses were conducted using Pearson’s correlation coefficients to examine the linear associations among the psychological variables of interest, including pain catastrophizing, perceived burdensomeness, hopelessness, suicidal ideation and childhood trauma.

These associations provided the empirical basis for testing the hypothesized mediation model. Subsequently, a multiple mediation analysis was performed to evaluate whether pain catastrophizing, perceived burdensomeness and hopelessness mediated the relationship between childhood trauma severity and suicidal ideation.

### 3.1. Correlations

Pearson correlations indicated positive and statistically significant associations among all variables included in the study. Childhood trauma showed small-to-moderate positive correlations with suicidal ideation (*r* = 0.332, *p* < 0.001), hopelessness (*r* = 0.224, *p* < 0.001), pain catastrophizing (*r* = 0.212, *p* < 0.001), and perceived burdensomeness (*r* = 0.266, *p* < 0.001).

Likewise, as depicted in Table 1, suicidal ideation was associated with moderate-to-strong levels of pain catastrophizing (*r* = 0.452, *p* < 0.001), perceived burdensomeness (*r* = 0.627, *p* < 0.001), and hopelessness (*r* = 0.546, *p* < 0.001).

Additionally, the mediator variables showed moderate positive associations with one another, with correlations between perceived burdensomeness and hopelessness (*r* = 0.524, *p* < 0.001), pain catastrophizing and hopelessness (*r* = 0.528, *p* < 0.001), and pain catastrophizing and perceived burdensomeness (*r* = 0.462, *p* < 0.001).

### 3.2. Mediational Analyses

**Figure 2:**
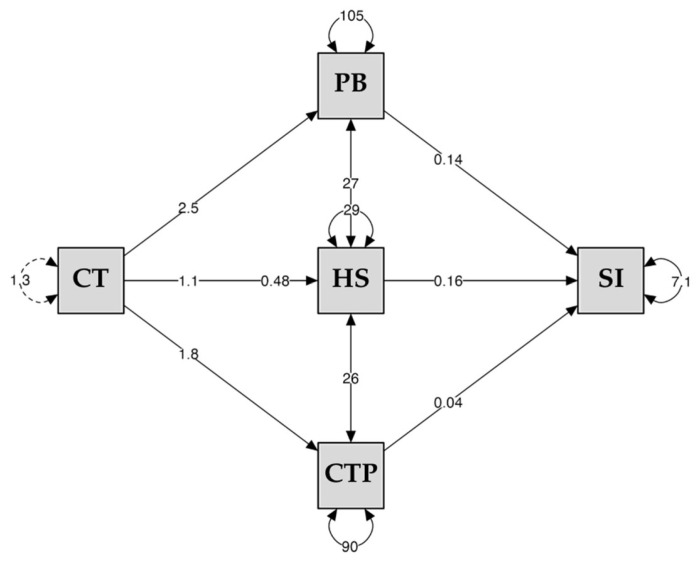
Multiple mediation model with the cognitive dimension from childhood trauma and suicidal ideation. Note. CT = Childhood Trauma, PB = Perceived Burdensomeness, HS = Hopelessness, CTP = Pain Catastrophizing, SI = Suicidal Ideation.

Table 2 and Figure 2 present the direct, indirect, and total effects of the proposed multiple mediation model.

The analysis revealed a significant total effect of childhood trauma on suicidal ideation (*β* = 0.480, 95% *CI* [0.154, 0.805], *p* = 0.004), as well as a significant direct effect when the mediators were included in the model (*β* = 1.091, 95% *CI* [0.672, 1.509], *p* < 0.001). Childhood trauma was significantly associated with perceived burdensomeness (*β* = 2.496, 95% *CI* [1.362, 3.629], *p* < 0.001), hopelessness (*β* = 1.095, 95% *CI* [0.470, 1.719], *p* < 0.001), and pain catastrophizing (*β* = 1.819, 95% *CI* [0.782, 2.855], *p* < 0.001). Each of these mediators showed a significant association with suicidal ideation (perceived burdensomeness: *β* = 0.145, 95% *CI* [0.103, 0.186], *p* < 0.001; hopelessness: *β* = 0.164, 95% *CI* [0.081, 0.247], *p* < 0.001; pain catastrophizing: *β* = 0.039, 95% *CI* [0.05, 0.72], *p* = 0.004 (see Figure 2).

The direct effect (c′) being greater than the total effect (c) is consistent with suppression patterns that can emerge when mediators share variance with both the predictor and the outcome. This configuration is common in multiple-mediator models and reflects overlapping explanatory pathways rather than an inconsistency in model specification.

The specific indirect effects were significant for perceived burdensomeness (*β* = 0.362, 95% *CI* [0.167, 0.558], *p* < 0.001), hopelessness (*β* = 0.179, 95% *CI* [0.042, 0.317], *p* = 0.011), and pain catastrophizing (*β* = 0.070, 95% *CI* [0.009, 0.139], *p* = 0.047). Consistently, the total indirect effect was likewise significant (*β* = 0.611, 95% *CI* [0.362, 0.869], *p* < 0.001) (see Table 2). Taken together, the indirect effects correspond to small-to-moderate magnitudes, consistent with partial rather than dominant mediation within the tested model. Among the mediators, perceived burdensomeness showed the largest indirect effect on suicidal ideation.

Overall, the pattern suggests a trajectory in which childhood trauma is associated with increased maladaptive cognitions, which in turn relate to greater suicidal ideation in this chronic pain population.

## 4. Discussion

Previous studies have highlighted the relevance of cognitive processes in the association between early adverse experiences and vulnerability to suicidal ideation [27,28]. In the present study, following this framework, two objectives were pursued: (1) to explore the associations among childhood trauma, perceived burdensomeness, hopelessness, pain catastrophizing, and suicidal ideation in individuals with chronic pain, and (2) to examine the mediating role of these cognitive processes in the relationship between childhood trauma and suicidal ideation through a multiple mediation model.

Regarding the first objective, the findings revealed positive and statistically significant associations among all variables analyzed, suggesting the presence of relevant psychological interactions in which early adversity and pain-related cognitive processes jointly contribute to the emergence of suicidal thoughts. This pattern aligns with prior literature [42,43]. However, our findings extend previous evidence by showing that, when examined simultaneously, perceived burdensomeness and hopelessness display stronger indirect associations with suicidal ideation than pain catastrophizing, suggesting differentiated cognitive pathways within the same model.

In this respect, childhood trauma was consistently related to suicidal ideation as well as with pain catastrophizing, hopelessness, and perceived burdensomeness. This pattern is congruent with previous research indicating that exposure to traumatic experiences during childhood constitutes one of the strongest predictors of suicide risk across the lifespan, both through its direct effects and through its contribution to the development of emotional and cognitive vulnerability schemas [44].

More specifically, among individuals with chronic pain, this association acquires relevance due to the high prevalence of early trauma in this population and the ways in which such experiences may shape cognitive interpretations, including alterations in the subjective experience of pain, perceptions of helplessness, and a persistent sense of threat [10,45].

Specifically, our results showed that suicidal ideation was most strongly associated with perceived burdensomeness and hopelessness, reinforcing their central role as mediating mechanisms in this sample. This finding aligns closely with the propositions of the Interpersonal Theory of Suicide, which posits that thoughts related to one’s own death arise fundamentally from the perception of being a burden to others and from the experience of interpersonal disconnection, especially when these perceptions are viewed as stable and unchangeable [32,43,46].

Moreover, individuals with chronic pain often face functional losses, reduced autonomy, and increasing dependence on other factors that may intensify the perception of generating an excessive emotional, economic, or caregiving burden within their close environment. When this feeling is combined with hopelessness, understood as the belief that current suffering will not change, it creates a scenario conducive to the development of suicidal ideation [47,48].

In turn, pain catastrophizing showed significant correlations with perceived burdensomeness, hopelessness, and suicidal ideation. The literature has consistently shown that catastrophizing may constitute one of the most representative predictors of psychological deterioration in patients with chronic pain [49]. It has also been linked to suicide risk due to its capacity to intensify perceived threat and increase rumination, as well as to generate a persistent sense of lack of control over daily functioning both present and future [42]. The fact that this variable shows moderate associations with hopelessness and perceived burdensomeness suggests that these cognitive processes may form part of shared processing and reality-integration schemas that amplify subjective distress and facilitate the emergence of suicidal ideation [10].

Regarding the second objective, the results reveal mediational relationships in which cognitive processes operate as potential explanatory mechanisms through which early trauma influences suicidal ideation among individuals with chronic pain.

In this context, pain catastrophizing showed the weakest indirect effect in the mediation model. This pattern may indicate that it functions primarily as a distress-amplifying variable that modulates, rather than directly determines, suicidal ideation. It may depend on other factors, such as depressive symptoms or pain intensity, to exert a stronger influence, which could explain its comparatively reduced contribution when examined alongside perceived burdensomeness and hopelessness.

First, perceived burdensomeness played a prominent mediating role. This finding aligns with previous research indicating that both early adverse experiences and illness-related situations may foster the emergence of beliefs related to worthlessness, guilt, or negative self-attributions, which tend to intensify in contexts of functional decline or actual dependence [12,15].

Within the field of chronic pain, such beliefs may progressively consolidate, especially when physical limitations and loss of autonomy increase the perception of generating relational strain with others. Additionally, recent evidence shows that the conviction of being a burden to loved ones may trigger a profound sense of mental defeat, understood as the perception of being completely overwhelmed by pain, which may further enhance the influence of this variable on suicide risk [50].

Likewise, hopelessness emerged as a potentially relevant mediator, aligning with contemporary theoretical proposals that position the cognitive framework as a central element in the transition from psychological pain to the desire to die. Recent literature indicates that, in individuals with chronic pain, this mediational relationship may be explained by the chronicity of physical discomfort, uncertainty regarding the progression of pain, and the possible accumulation of therapeutic failures all of which reinforce the belief that the situation will not improve, particularly among those who carry early negative cognitive schemas derived from childhood trauma [10,51,52].

Thus, hopelessness would represent a significant mediator between physical pain and thoughts of one’s own death, both in clinical and non-clinical populations, suggesting that early adverse experiences and pain may foster a pessimistic outlook on the future, thereby contributing to an increased risk of suicide [53].

Regarding pain catastrophizing, recent research has indicated that early traumatic experiences may orient affected individuals toward processing styles characterized by threat amplification, difficulties in emotional regulation, and heightened vulnerability to stress [54,55]. Specifically, among individuals with chronic pain, these cognitive patterns not only persist but tend to intensify, leading to exaggeratedly negative interpretations of painful sensations, increased rumination, and a persistent perception of helplessness in the face of psychological and physical suffering. This cluster of processes may configure a risk profile that may indirectly contribute to the development of suicidal ideation, with catastrophizing functioning as a mechanism through which the impact of childhood trauma manifests in thoughts related to suicide [56].

In parallel, this threatening interpretation of the pain experience is associated with greater distress and increased pain sensitivity circumstances reinforced by cognitive difficulties in modulating emotional responses [57,58]. Ultimately, these dynamics foster the emergence of psychiatric comorbidity, including suicidal ideation, and contribute to a significant deterioration in quality of life [49].

Our findings contribute additional evidence by testing these mediators jointly in a chronic pain population, a focus that, to our knowledge, has not been examined in previous studies. Taken together, the present study suggests that the cognitive dimension may play a meaningful role in the relationship between childhood trauma and suicidal ideation within the context of chronic pain, specifically through perceived burdensomeness, hopelessness, and pain catastrophizing. These results indicate that early adverse experiences influence both how individuals interpret suffering and how they cope with the changes associated with persistent pain, thereby fostering cognitive patterns that heighten psychological vulnerability [49,59,60]. From this integrative perspective, the interaction between life history, chronic pain, and maladaptive cognitions may contribute to a more comprehensive understanding of the complexity of suicide risk [60,61].

This pattern, in which all mediation paths reached significance, can be interpreted through a transdiagnostic lens. In chronic pain, trauma history, maladaptive cognitions, and suicidal ideation frequently operate as overlapping vulnerability processes rather than isolated predictors, which may explain the concurrent significance of the mediators. This interpretation is consistent with models describing chronic pain as a condition in which cognitive, emotional, and interpersonal factors interact dynamically in the configuration of psychological suffering.

These findings point to several potential implications for assessment and intervention regarding suicide risk in individuals with chronic pain. On the one hand, they suggest the relevance of incorporating systematic assessment of childhood trauma in healthcare services treating patients with persistent pain, given that such trauma may represent an important vulnerability factor for both the pain experience and the development of suicidal ideation. Likewise, the results indicate that variables such as pain catastrophizing, hopelessness, and perceived burdensomeness could be explicitly integrated into suicide risk screening protocols, since elevated levels of these variables may function as warning signs that warrant prompt clinical attention. Finally, these factors (together with early adverse experiences) could be considered potential targets for psychological intervention, particularly in programs focused on cognitive restructuring, reduction in emotional distress, and suicide prevention among patients with chronic pain. Therefore, this study provides an integrated cognitive framework that may inform trauma-informed clinical assessment and guide intervention planning in chronic pain settings, without assuming causal precedence or overgeneralizing the mediation patterns observed.

Despite the strengths of the study, several limitations should be considered, as they nuance the interpretation of the findings and inform future research directions. The cross-sectional design precludes causal inferences, underscoring the need for longitudinal approaches capable of clarifying the temporal sequencing of childhood trauma, maladaptive cognitions, and suicidal ideation. Additionally, the use of self-report measures introduces susceptibility to memory biases and social desirability, particularly in the assessment of childhood trauma and suicidality, highlighting the value of complementary and multimethod procedures. The exclusive reliance on self-report instruments for sensitive constructs may further increase vulnerability to reporting bias, and the dichotomous assessment of suicidal ideation may restrict variability and underestimate effect sizes, supporting the use of continuous measures in future studies. The overrepresentation of women in the sample (84.5%) may also limit generalizability; however, this imbalance likely reflects common patterns of healthcare utilization in chronic pain services rather than a methodological sampling bias. Finally, the model focused exclusively on cognitive mediators, without including emotional or spiritual dimensions that may contribute to a more comprehensive understanding of the phenomenon.

Building on these considerations, future research should prioritize longitudinal and prospective designs to clarify developmental pathways linking trauma exposure, cognitive vulnerability, and suicidal ideation in chronic pain populations. Such studies would benefit from multimethod assessment strategies integrating self-report, clinician-rated, behavioral, and physiological indicators, particularly when examining sensitive constructs. Stratified recruitment approaches are also warranted to ensure more balanced sex distributions and to explore potential sex-specific pathways in pain processing and suicide risk. Moreover, the use of dimensional and continuous measures of suicidal ideation may enhance sensitivity and improve the precision of effect size estimation. Finally, extending the current cognitive mediation framework to incorporate emotional, interpersonal, and existential domains, alongside beneficially adaptive cognitions such as coping resources, perceived social support, and self-efficacy, may facilitate the development of more comprehensive, trauma-informed, and prevention-oriented models of suicide risk in individuals with chronic pain.

## 5. Conclusions

The findings of this study suggest that cognitive processes (particularly perceived burdensomeness, hopelessness, and pain catastrophizing) may play a relevant role in the association between childhood trauma and suicidal ideation in individuals with chronic pain. This potential mediating pathway underscores the usefulness of considering both early adverse experiences and pain-related cognitive patterns in the comprehensive assessment of suicide risk within this specific clinical population. Overall, these results provide preliminary support for an integrated cognitive-interpersonal model that identifies modifiable intervention targets and may inform trauma-informed approaches in chronic pain care.

Likewise, the results highlight the importance of continuing to examine these cognitive mechanisms within therapeutic settings, as they may represent valuable intervention targets for reducing psychological vulnerability and improving the clinical management of persistent pain.

Nevertheless, considering the methodological limitations of the study, future research should employ longitudinal designs, multimethod assessment strategies, and more diverse and stratified chronic pain samples. Such approaches would allow for greater precision in clarifying the nature and scope of these associations and would support the development of integrated explanatory models of suicidal ideation in the context of chronic pain, thereby improving generalizability and clinical applicability.

## Figures and Tables

**Table 1:** Correlations among the study variables.

Variable	1	2	3	4	5
CT	—				
2. PB	0.266 ***	—			
3. HS	0.224 ***	0.524 ***	—		
4. CTP	0.212 ***	0.462 ***	0.528 ***	—	
5. SI	0.332 ***	0.627 ***	0.546 ***	0.452 ***	—

Note. *** *p* < 0.001; CT = Childhood Trauma, PB = Perceived Burdensomeness, HS = Hopelessness, CTP = Pain Catastrophizing, SI = Suicidal Ideation.

**Table 2:** Direct, indirect, and total effects of the multiple mediation model.

Effect	Trajectory	*β*	95% *CI*	*p*
**Direct Effects**				
Total effect (c)	CT → SI	0.480	[0.154, 0.805]	0.004
Direct effect (c’)	CT → SI			
	(mediators controlled)	1.091	[0.672, 1.509]	<0.001
**Path Mediation (a)**				
a1	CT → PB	2.496	[1.362, 3.629]	<0.001
a2	CT →HS	1.095	[0.470, 1.719]	<0.001
a3	CT → CTP	1.819	[0.782, 2.855]	<0.001
**Path Mediation (b)**				
b1	PB → SI	0.145	[0.103, 0.186]	<0.001
b2	HS → SI	0.164	[0.081, 0.247]	<0.001
b3	CTP → SI	0.039	[0.05, 0.72]	0.004
**Specific indirect effects**				
ab1	CT →PB → SI	0.362	[0.167, 0.558]	<0.001
ab2	CT → HS → SI	0.179	[0.042, 0.317]	0.011
ab3	CT → CTP → SI	0.070	[0.009, 0.139]	0.047
**Total indirect effects**				
	CT → Mediators → SI	0.611	[0.362, 0.869]	<0.001

Note. CT = Childhood Trauma, PB = Perceived Burdensomeness, HS = Hopelessness, CTP = Pain Catastrophizing, SI = Suicidal Ideation.

## Data Availability

On request to the corresponding author.
